# Clinical Value of Amide Proton Transfer-weighted Imaging in Ischemic Stroke: A Comparative Study with Apparent Diffusion Coefficient and CT Perfusion

**DOI:** 10.2174/0115734056493222260804100143

**Published:** 2026-08-27

**Authors:** Le Zhou, Renjun Huang, Hui Wang, Mengying Zhu, Xiaofei Zhou, Yi Zhang, Xinbing Zheng, Yonggang Li

**Affiliations:** 1 Department of Radiology, The First Affiliated Hospital of Soochow University, Suzhou City, Jiangsu Province, 215006, China; 2 Department of Neurology, The First Affiliated Hospital of Soochow University, Suzhou, Jiangsu, 215000, China; 3 Key Laboratory for Biomedical Engineering of Ministry of Education, Department of Biomedical Engineering, College of Biomedical Engineering and Instrument Science, Zhejiang University, Hangzhou, Zhejiang, China; 4 Department of Radiology, Zhangjiagang Sixth People's Hospital, Suzhou City, Jiangsu Province, 215600, China; 5 Institute of Medical Imaging, Soochow University, Suzhou City, Jiangsu Province, 215000, China

**Keywords:** CEST, APT, Ischemic stroke, Magnetic resonance imaging, Computed tomography, Prognostic prediction

## Abstract

**Introduction:**

Diffusion- and perfusion-based imaging, including Apparent Diffusion Coefficient (ADC) and CT Perfusion (CTP), are standard tools for evaluating ischemic stroke but primarily reflect structural and hemodynamic changes. They provide limited insight into tissue metabolism. Amide Proton Transfer-weighted (APTw) imaging enables noninvasive assessment of pH-related metabolic alterations and may offer complementary diagnostic and prognostic information.

**Materials and Methods:**

In this prospective study, 54 patients with hyperacute or acute ischemic stroke (mean age 64.39 ± 10.91 years; 41 males, 13 females) were enrolled. Correlations between APTw and conventional imaging parameters, including ADC, Cerebral Blood Volume (CBV), Cerebral Blood Flow (CBF), and Time to Peak (TTP), were analyzed. Kaplan–Meier analysis assessed 18-month outcomes. Time-dependent Receiver Operating Characteristic (ROC) analysis, decision curve analysis (DCA), calibration curves, and Clinical Impact Curves (CIC) were used to evaluate prognostic performance.

**Results:**

APTw values were significantly correlated with CBV (r = 0.6886), CBF (r = 0.6702), ADC (r = 0.6565), and TTP (r = –0.6519) (all *p* < 0.05). Kaplan–Meier analysis demonstrated significant differences in 18-month outcomes between APTw-based subgroups (log-rank *p* < 0.05). APTw achieved higher AUCs at 6, 12, and 18 months (0.821, 0.831, and 0.877) than ADC and CTP parameters. DCA, calibration, and CIC analyses confirmed stable predictive performance.

**Discussion:**

APTw showed strong concordance with diffusion- and perfusion-based biomarkers while providing complementary metabolic information, suggesting exploratory comparative prognostic relevance in ischemic stroke.

**Conclusion:**

APTw imaging offers complementary metabolic insight and may enhance diagnostic and prognostic evaluation in ischemic stroke.

## 1 INTRODUCTION

Ischemic stroke, a leading cause of disability and death worldwide, is characterized by the interruption of blood flow to specific brain regions, resulting in rapid and severe impairment of neurological function, and accounting for the majority of stroke cases [1]. The underlying pathophysiology involves blockage of cerebral arteries by blood clots or emboli, leading to inadequate perfusion, ischemia, and eventual infarction of brain tissue [2]. Thus, timely and accurate diagnosis of ischemic stroke is critical for effective treatment and improving patient prognosis [3].

Among current neuroimaging modalities, Magnetic Resonance Imaging (MRI) and Computed Tomography (CT) are the two principal techniques used for acute stroke assessment. Within MRI, diffusion-weighted imaging and its derived Apparent Diffusion coefficient (ADC) quantify water molecule diffusion restriction and primarily reflect cytotoxic edema. In contrast, CT Perfusion (CTP), a CT-based functional imaging technique, evaluates cerebral hemodynamics through parameters such as Cerebral Blood Flow (CBF), Cerebral Blood Volume (CBV), and Time to Peak (TTP), thereby characterizing perfusion deficits.

While both are essential tools in stroke assessment, they have some limitations. ADC is useful for detecting early ischemic changes but is primarily sensitive to cytotoxic edema, making it less effective in distinguishing irreversibly infarcted tissue from the ischemic penumbra, particularly in cases of mixed or junctional ischemia [4]. Similarly, CTP offers valuable insights into cerebral perfusion dynamics, including the identification of the ischemic penumbra and infarct core. However, its accuracy can be influenced by factors such as contrast injection timing, patient motion, and poor image quality, particularly in cases involving severe edema or extensive stroke. Additionally, the dependence of CTP on Arterial Input Function (AIF) estimates can result in inaccurate perfusion metrics. These limitations highlight the need for alternative imaging techniques that can offer more comprehensive and reliable information [5, 6].

In recent years, Amide Proton Transfer-weighted (APTw) imaging has garnered significant attention as an emerging Magnetic Resonance Imaging (MRI) technique. As a key component of Chemical Exchange Saturation Transfer (CEST) imaging, APTw imaging indirectly reflects tissue metabolism by detecting the exchange of protons between amide groups in proteins and peptides and water protons [7].

Previous studies have emphasized the significant role of APTw imaging in diagnosing Central Nervous System (CNS) diseases. In the field of neuro-oncology, it has demonstrated utility in differentiating tumor recurrence from radionecrosis and in noninvasively grading gliomas without the need for contrast agents [8, 9]. Beyond oncology, APTw imaging has shown promise in ischemic stroke, with studies reporting its efficacy in predicting infarct size and extent [10], its inverse relationship with clinical symptom severity [11], and its demonstrated sensitivity to hemorrhagic lesions in related neurovascular applications [12]. These capabilities suggest that APTw imaging provides complementary metabolic information to conventional diffusion- and perfusion-based techniques [13].

Early identification of subtle metabolic disturbances may provide clinically relevant information beyond structural infarct characterization, potentially facilitating improved risk stratification and individualized therapeutic decision-making [14].

This study aimed to evaluate the correlation of APTw imaging with ADC and CTP parameters, and to further explore its metabolic value and predictive stability in assessing outcomes in ischemic stroke.

## 2 MATERIALS AND METHODS

### 2.1 Study Population

This prospective study was approved by the local Institutional Review Board (IRB), and all participants provided written informed consent prior to enrollment.

A total of 72 patients with hyperacute and acute ischemic stroke were prospectively enrolled at First Affiliated Hospital of Soochow University, China between September 2021 and August 2023. After applying the enrollment criteria, 54 patients were ultimately included in this study (Fig. **1**).

Inclusion criteria were as follows: 1) onset of neurological symptoms and MRI and CT evaluation within 72 hours, with an interval between MRI and CTP examinations not exceeding 6 hours; and 2) no prior endovascular treatment before imaging. The exclusion criteria were as follows: 1) age < 18 years; 2) infarct lesion diameter < 10 mm on diffusion-weighted imaging (DWI); 3) presence of intracranial neoplasms; 4) history of ischemic stroke; 5) suboptimal image quality; and 6) other concurrent brain disorders.

Image quality was assessed by Yonggang Li (21 years of experience) and Renjun Huang (11 years of experience), demonstrating strong inter-rater agreement (intraclass correlation coefficient [ICC] = 0.887, 95% confidence interval [CI] = 0.814–0.932).

At admission, a neurologist (Hui Wang, with 20 years of experience in stroke diagnosis and treatment) assessed patients using the National Institutes of Health Stroke Scale (NIHSS), and follow-up evaluations were conducted for 18 months post-discharge, including a modified Rankin Scale (mRS) score. An mRS score of ≤ 2 was considered a good prognosis, while an mRS score > 2 indicated a poor prognosis. During follow-up, information regarding patient mortality or recurrence was also collected. The overall study design and imaging assessment framework are summarized in Fig. (**2**).

### 2.2 CT Acquisition

Imaging was performed on a GE Revolution CT system (GE Healthcare, Wisconsin, USA) prior to endovascular treatment and included non-enhanced CT (NECT), CT Angiography (CTA), and CTP.

#### 2.2.1 NECT

Acquisition parameters included 120 kVp, 320 mAs, a 1.25-mm slice thickness, and a 512 × 512 matrix. CTP Imaging was conducted at 100 kVp and 150 mAs, with a 5-mm slice thickness and a 512 × 512 matrix. Data acquisition spanned 60 seconds, capturing 20 phases at 3-second intervals. CTA: A multiphase protocol utilized rapid dual-kVp (80 and 140) switching, 320 mAs, and a 0.625-mm slice thickness with a 512 × 512 matrix. The initial phase included aortic arch-to-vertex imaging, while subsequent phases covered the skull base to the vertex during the equilibrium/peak venous and late venous phases. Following table repositioning, the second phase began after a 4-second delay, with subsequent phases lasting 3.4 seconds each. For contrast-enhanced imaging, 40 mL of iodine-based contrast agent was administered intravenously at 5 mL/s, followed by a 50 mL saline flush.

### 2.3 MRI Acquisition

The mean interval between CTP and MRI acquisition was 50.6 ± 10.4 minutes. MRI data were acquired using a Siemens 3T Skyra system (SIEMENS, Erlangen, Germany) equipped with an 8-channel head coil. Standard sequences included T1-Weighted Imaging (T1WI), T2-Weighted Imaging (T2WI), T2-fluid-attenuated inversion recovery (T2-FLAIR), DWI, and Magnetic Resonance Angiography (MRA). Sequence parameters were as follows: T1WI: FOV = 230 × 230 mm^2^, TR = 220 ms, TE = 2.46 ms; T2WI: FOV = 220 × 220 mm^2^, TR = 3700 ms, TE = 93 ms; T2-FLAIR: FOV = 220 × 220 mm^2^, TR = 7000 ms, TE = 78 ms; DWI: FOV = 220 × 220 mm^2^, TR = 4330 ms, TE = 61 ms, b-values = 0 and 1000 s/mm^2^; MRA: FOV = 200 × 180.4 mm^2^, TR = 21 ms, TE = 3.43 ms.

CEST imaging was subsequently performed at the level of the largest infarct area identified on DWI, as determined by an experienced radiologist (Le Zhou, with 6 years of expertise). The CEST imaging protocol [15, 16] comprised four primary modules: frequency stabilization, saturation, fat suppression, and readout. Saturation involved the application of ten 100-ms Gaussian pulses, separated by 5-ms, 10-mT/m crusher gradients.

The readout utilized a turbo spin-echo sequence with the following parameters: TR = 3000 ms, TE = 7.2 ms, FOV = 211 × 185.5 mm^2^, matrix size = 96 × 84, turbo factor = 84, and slice thickness = 5 mm. Single-slice images were acquired at a far off-resonant frequency (>1500 ppm) for normalization (S0) and at 62 frequency offsets: 0, ±0.25, ±0.5, ±0.75, ±1, ±1.5, ±2 (2), ±2.5 (2), ±3 (2), ±3.25 (2), ±3.5 (6), ±3.75 (2), ±4 (2), ±4.5, ±5, ±6, 10, 15.65, 20, 30, 40, 50, 60, 70, and 80 ppm (numbers in parentheses indicate repeats). CEST datasets were acquired using three saturation powers (1, 1.5, and 2 μT), each lasting 3.2 minutes. B0 and B1 maps were generated at the same spatial location to facilitate correction and analysis [17]. The acquisition time of the APTw sequence was approximately 10 minutes.

### 2.4 Image Processing and Analysis

Post-processing of the acquired CEST images was performed using MATLAB (Version R2022b, MathWorks, Natick, MA, USA). The magnetization transfer ratio asymmetry (MTR_asym_, also known as APTw) was calculated using the following formula [7, 15]:


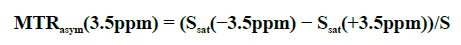


Regions of Interest (ROIs) were independently delineated by two neuroradiologists (Renjun Huang and Le Zhou, with 11 and 6 years of experience, respectively) who were blinded to NIHSS and mRS scores. The slice containing the largest infarct diameter, identified on DWI, was selected for analysis. Up to three ROIs were placed within the lesion, first drawn on the DWI images and then transferred to APTw images. The lowest signal value within the infarcted lesion, termed APTmin, was recorded. APTmin was selected because the lowest focal APTw signal is presumed to represent the region of greatest metabolic derangement and tissue acidosis within the ischemic lesion, potentially providing the most sensitive indicator of irreversible injury. Corresponding ADC values were also collected from the same slice for comparative analysis.

For hemodynamic assessment, CTP images were initially processed using MIStar software (Apollo Medical Imaging Technology, Australia). This software applies the central volume principle to calculate core perfusion metrics, including cerebral Blood Flow (CBF), derived from the peak height of the impulse residue function curve; Cerebral Blood Volume (CBV), calculated as the area under the curve; and Mean Transit Time (MTT), computed as the CBV-to-CBF ratio. Delay Time (DT) was calculated with delay and dispersion correction.

Based on previously validated thresholds implemented in MIStar, Perfusion Lesions (PL) were defined as regions with DT > 3 s, and Infarct Cores (IC) were identified as areas with CBF < 30% within regions of DT > 3 seconds.

The MIStar-processed perfusion datasets were subsequently imported into the Brain Perfusion application on the Intellispace Portal (Philips Healthcare, Netherlands) for standardized visualization and extraction of additional perfusion parameters, including Time to Peak (TTP). These parameters were used for comparative and correlation analyses only and did not affect the definition of PL or IC. Hemodynamic parameters were analyzed bilaterally in both the infarcted and contralateral hemispheres.

### 2.5 Clinical Outcome Definitions

Two clinically distinct outcome endpoints were evaluated in this study. Functional outcome was assessed using the modified Rankin Scale (mRS) at 6, 12, and 18 months after stroke onset. Poor functional outcome was defined *a priori* as mRS >2 (binary classification) and served as the endpoint for time-dependent ROC analyses, decision Curve Analysis (DCA), calibration curve analysis, and Clinical Impact Curve (CIC) assessment. In addition, the time-to-event outcome was defined as the composite endpoint of first recurrent ischemic stroke or all-cause mortality, whichever occurred first, during the entire 18-month follow-up period. Events were prospectively documented throughout follow-up as they occurred, rather than being restricted to scheduled follow-up assessments. Time from stroke onset to the first composite event was used for Kaplan–Meier analysis of event-free survival. Patients who remained free of either event were censored at the time of their last available clinical follow-up, with administrative censoring at 18 months when complete follow-up was available.

### 2.6 Statistical Analysis

Statistical analyses were conducted using SPSS 26.0 (IBM Corporation, Armonk, NY, USA). and R software (version 4.4.1). The Kolmogorov-Smirnov test was applied to evaluate the normality of quantitative data and determine the appropriateness of subsequent statistical methods. Continuous variables with normal distributions were expressed as mean ± standard deviation and compared using independent-samples t-tests. Non-normally distributed continuous variables were presented as medians with Interquartile Ranges (IQR) and compared using the Mann-Whitney U test. Categorical variables were summarized as frequencies and percentages, with between-group comparisons performed using the chi-square test. Although several imaging variables were descriptively presented as median (interquartile range) for subgroup comparisons, normality and linearity of full-cohort imaging parameter distributions were separately assessed prior to correlation analysis. Based on these assessments, Pearson correlation analysis was used to evaluate linear relationships between imaging parameters. Time-dependent Receiver Operating Characteristic (ROC) analyses were performed to evaluate the prognostic discrimination of imaging biomarkers for poor functional outcome, defined as modified Rankin Scale (mRS) >2, at 6, 12, and 18 months after stroke onset. Formal pairwise comparison of ROC curves using DeLong’s test and multivariable incremental discrimination analyses (*e.g*., net reclassification improvement and integrated discrimination improvement) were not performed because the limited sample size would likely render such analyses underpowered and potentially unstable, which was considered inappropriate for this exploratory study. Kaplan-Meier survival analyses were conducted using the composite time-to-event endpoint of recurrent ischemic stroke or all-cause mortality during continuous 18-month follow-up. Patients without events were censored at the time of their last available follow-up. Decision Curve Analysis (DCA), calibration curves, and Clinical Impact Curves (CIC) were performed using single-predictor logistic regression models, with 18-month poor functional outcome (mRS >2) as the binary outcome variable. Each imaging biomarker was evaluated separately in an exploratory comparative framework to assess relative prognostic behavior rather than to establish or validate a multivariable clinical prediction model. A two-sided *p* value < 0.05 was considered statistically significant.

## 3 RESULTS

### 3.1 Baseline Information

According to the enrollment criteria, 54 patients with hyperacute and acute ischemic stroke (mean age 64.39 ± 10.91 years; 41 males, 13 females) were included in this study (Table **1**). For the KM survival analysis, patients were classified into two groups: Low APTw (n = 29) and High APTw (n = 25) based on the median APTmin, as well as Low APTw_1_ (n = 21) and High APTw_1_ (n = 33) based on the ROC cutoff value.

### 3.2 Clinical Outcomes

At 6 months, 3 patients (5.6%) had poor functional outcome (mRS >2), compared with 7 (13.0%) at 12 months and 17 (31.5%) at 18 months. During the continuous 18-month follow-up period, 34 patients (63.0%) experienced recurrent ischemic stroke and 11 (20.4%) died, yielding a total of 45 composite events (83.3%).

### 3.3 Correlation of APTmin with ADC and CTP Parameters

Pearson correlation analysis revealed that APTmin was correlated with ADC (r = 0.6565, 95% CI: 0.4838 – 0.7794), IC-CBV (r = 0.6886, 95% CI: 0.5284 – 0.8015), IC-CBF (r = 0.6702, 95% CI: 0.5033 –0.7889), and IC-TTP (r = -0.6519, 95% CI: -0.7763 – -0.4786). Among these, the strongest association was observed between APTmin and IC-CBV (r = 0.6886, R^2^ = 0.4742, *P* < 0.0001) (Fig. **3**).

### 3.4 Kaplan-Meier Analysis of APTmin Subgroups

Patients were stratified into two groups based on the optimal cutoff value of −1.514 determined by ROC analysis (Fig. **4a**, **b**), resulting in a low APTw_1_ group (n = 21) and a high APTw_1_ group (n = 33) (Fig. **4c**). To assess the robustness of the survival analysis, patients were additionally classified into low (n = 29) and high (n = 25) APTw groups using the median APTmin value as an alternative threshold (Fig. **4d**). Kaplan–Meier survival analysis showed differences in survival and recurrence between subgroups over the 18-month follow-up period, as assessed by the log-rank test (*P* < 0.05).

### 3.5 Time-Dependent ROC Analysis

Dynamic time-dependent ROC analyses were performed to evaluate the association between APTmin, ADC, CBV, CBF, and TTP and patient outcomes at 6, 12, and 18 months (Fig. **5**). Among these parameters, APTmin showed favorable discrimination ability at each time point, with Area Under the Curve (AUC) values of 0.821, 0.831, and 0.877 at 6, 12, and 18 months, respectively.

### 3.6 Internal Validation of Imaging Biomarkers

Evaluation of the time-dependent ROC–based analyses presented in Fig. (**5**) was further performed using DCA, calibration curves, and CIC. Detailed ROC results are provided in Table **S1**. As shown in Fig. (**6**), analyses based on APTmin suggested stable performance and relatively higher net benefit across a range of threshold probabilities compared with those based on other imaging biomarkers. Figures **7** and **8** depict stroke cases.

## 4 DISCUSSION

This study demonstrated significant correlations between APTw and ADC, CBV, CBF, and TTP. Kaplan–Meier survival analysis showed differences in 18-month outcomes between patient subgroups stratified by APTw values. Time-dependent ROC analysis further indicated that APTw exhibited favorable discrimination ability across multiple time points compared with other imaging parameters. Finally, the stability and decision performance of each parameter were further evaluated. Unlike conventional diffusion and perfusion imaging, which primarily reflect structural restriction and hemodynamic compromise, APTw may capture tissue-level metabolic disturbance, potentially explaining its distinct metabolic sensitivity and its possible complementary prognostic relevance. Compared with prior studies primarily focused on short-term outcome prediction or isolated MRI-based comparisons, the present work contributes a multimodal comparative framework integrating APTw, ADC, and CT perfusion metrics, while extending prognostic evaluation to long-term follow-up.

ADC serves as a crucial biomarker for evaluating tissue viability in acute ischemic stroke, with lower values typically indicating more severe injury and poorer outcomes [18–23]. CTP imaging utilizes parameters such as CBF, CBV, and MTT to estimate the ischemic penumbra and infarct core [24]. Among these, CBF serves as a direct quantitative marker of tissue perfusion and metabolic supply. Normal neuronal function requires a minimum CBF of approximately 20 mL/100 g/min; CBV reflects the capillary bed volume and collateral circulatory reserve. During ischemia, local vasodilation and collateral recruitment may maintain or slightly increase CBV in the penumbra, whereas markedly decreased CBV is observed in the infarct core due to loss of autoregulatory capacity. TTP is commonly used to detect perfusion delays; prolonged TTP generally reflects impaired arterial inflow or increased collateral flow pathways [6, 25, 26].

In this study, APTw imaging showed good correlations with ADC, CBF, CBV, and TTP, potentially reflecting the metabolic alterations-including anaerobic glycolysis and tissue acidification-occurring in hypoperfused regions. Prior research has similarly demonstrated that APTw imaging is sensitive to ischemia-induced metabolic changes [27]. Although APTw, ADC, and CTP differ in physiological basis, all three can identify ischemic tissue and provide complementary diagnostic information.

Our findings are broadly consistent with prior studies demonstrating the prognostic relevance of APT-based imaging in ischemic stroke. Yu *et al*. reported that reduced APTw signal was associated with poorer functional recovery in patients receiving supportive treatment [11]. At the same time, He *et al*. showed that combining APT imaging with arterial spin labeling improved prediction of post-thrombolysis outcomes [13]. The present study extends these observations in several important ways. First, we directly compared APTw with both diffusion-derived and CT perfusion-derived parameters within the same patient cohort. Second, unlike most previous studies focused on short-term outcomes (typically 90 days), we evaluated prognostic performance over an 18-month follow-up period. Third, our findings suggest that metabolically sensitive APTw imaging may provide prognostic information beyond conventional structural and hemodynamic biomarkers, supporting its potential as an exploratory complementary imaging biomarker for longitudinal stroke assessment.

Guo *et al*. evaluated the prognostic value of ADC in 94 patients with acute posterior circulation stroke treated with endovascular therapy. Using ROC analysis, they identified an optimal ADC cutoff with an AUC of 0.796 for predicting 3-month outcomes [18]. In our study, ADC was also applied to assess prognosis at 6, 12, and 18 months, yielding AUCs of 0.724, 0.611, and 0.522, respectively. In contrast, APTw showed favorable prognostic performance, with AUCs of 0.821, 0.831, and 0.877 at the same time points, which may be related to its sensitivity to metabolic disturbances and tissue acidosis in ischemic regions [27–29].

Similarly, Hoving *et al*. used core volume derived from CTP to predict 90-day outcomes, reporting an AUC of 0.802, supporting the prognostic value of perfusion imaging [30]. In our study, among CTP-derived parameters, CBV had the highest AUC at 6 months (0.773), while TTP achieved the highest AUC at both 12 (0.811) and 18 months (0.771); whereas APTw yielded numerically higher ROC estimates in this exploratory cohort. Although methodological and cohort differences prevent direct comparison, these findings further support the potential prognostic relevance of APTw in ischemic stroke.

Moreover, exploratory decision-analytic analyses suggested relatively consistent comparative performance of APTw across evaluated imaging biomarkers. These findings suggest that APTw may provide complementary insights into tissue damage, ischemic penumbra, and perfusion deficits, thereby suggesting potential complementary relevance for exploratory prognostic assessment.

Although APTmin demonstrated numerically favorable ROC estimates compared with several conventional imaging biomarkers, formal statistical comparison of ROC curves using DeLong’s test was not performed, and no multivariable incremental modeling incorporating ADC or CTP-derived parameters was constructed. Therefore, the present findings should not be interpreted as evidence that APTw provides statistically superior or additive prognostic value beyond established diffusion- and perfusion-based imaging techniques. Rather, these observations should be regarded as preliminary comparative findings that warrant further investigation in larger cohorts using formal incremental discrimination analyses, including Net Reclassification Improvement (NRI) and Integrated Discrimination Improvement (IDI) analyses.

In this study, KM curves were generated based on APTw imaging. The analysis revealed differences in mortality and recurrence rates at 18 months across patient subgroups, as assessed by the log-rank test (*P* < 0.05), under both ROC-derived and median-based stratification strategies. These findings further suggest the exploratory prognostic relevance of APTw as an imaging biomarker and are broadly consistent with prior observational reports [11, 13]. These exploratory analyses should not be interpreted as formal model validation but rather as comparative assessments of relative biomarker performance. It should be noted that functional outcome analyses and event-free survival analyses addressed distinct clinical endpoints, reflecting complementary but non-identical aspects of long-term stroke prognosis.

## 5 STUDY LIMITATIONS

Despite the promising findings of this study, several limitations should be acknowledged. First, prognostication after acute ischemic stroke in real-world clinical practice relies on a multidimensional framework that integrates imaging biomarkers with neurological severity, premorbid functional status, vascular risk burden, treatment response, and systemic medical factors. Owing to the relatively small sample size, we were unable to perform robust multivariable prognostic modeling incorporating established clinical covariates such as baseline NIHSS score, lesion size, and demographic characteristics. Consequently, although APTw demonstrated favorable comparative prognostic discrimination in this cohort, potential confounding effects cannot be excluded, and the observed predictive performance may have been overestimated. While our previous work demonstrated that APTmin was an independent predictor of 90-day functional outcome after multivariable adjustment, the present findings should be considered exploratory and hypothesis-generating. Independent validation in larger cohorts using integrated clinical-imaging prognostic models will be essential to determine the true incremental value of APTw.

Second, this was a single-center study performed using a single scanner model and field strength, which may limit the generalizability of the findings across different vendors, acquisition protocols, and clinical settings, thereby necessitating further multicenter validation. In addition, the present analysis focused exclusively on APTmin. Other potentially informative APTw-derived metrics, including mean, median, maximum, and volumetric histogram-based parameters, were not evaluated and may provide complementary prognostic information in future studies.

Third, APTmin was derived from manually delineated ROIs on a single representative slice. Although strong inter-observer agreement was observed, this two-dimensional sampling strategy remains inherently operator-dependent and may not fully capture the three-dimensional spatial heterogeneity of ischemic lesions, particularly in larger or morphologically complex infarcts. Consequently, some degree of sampling bias cannot be excluded, which may limit the broader applicability of APTw imaging for comprehensive lesion assessment.

Fourth, the optimal APTmin threshold was both derived and evaluated within the same cohort. This same-sample validation approach is inherently susceptible to optimism bias and may artificially inflate the apparent prognostic discrimination of the proposed cut-off. Because of the limited sample size, neither internal split-sample validation nor external validation was statistically feasible. Therefore, the proposed threshold should be regarded as preliminary and requires confirmation through formal internal resampling approaches (*e.g*., bootstrap validation) and independent external cohort validation.

## CONCLUSION

APTw imaging may provide complementary metabolic information for prognostic assessment in ischemic stroke. However, the present findings are exploratory and should not be interpreted as evidence of definitive superiority or additive prognostic value beyond established imaging biomarkers. Larger multicenter studies incorporating formal incremental discrimination analyses and external validation will be essential to determine its true clinical utility.

## Figures and Tables

**Fig. (1):**
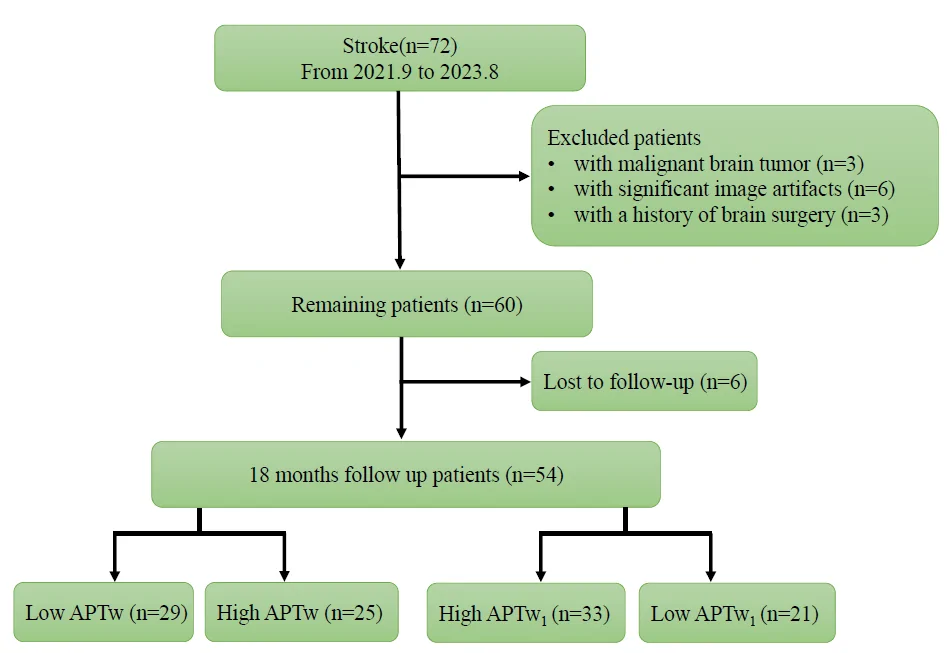
Patient selection flowchart.

**Fig. (2):**
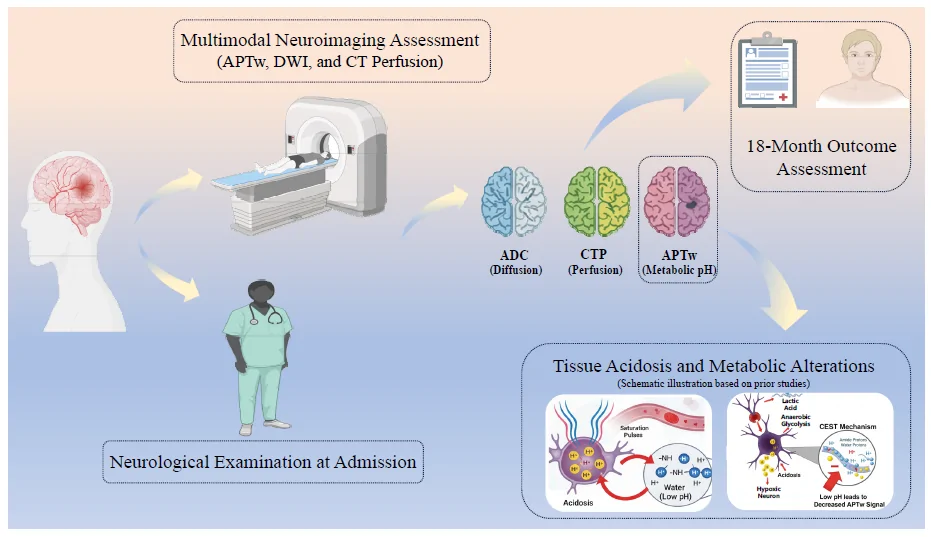
Study design and conceptual framework of multimodal neuroimaging assessment in ischemic stroke.

**Fig. (3):**
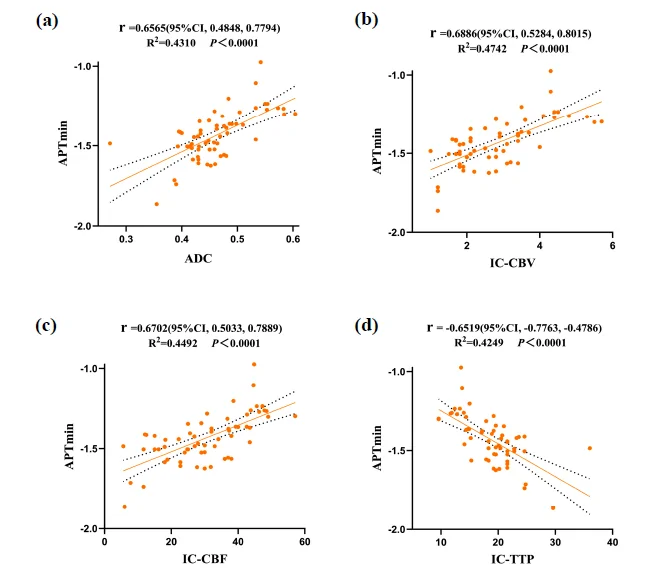
Correlation of APTw with (**a**) ADC, (**b**) IC-CBV, (**c**) IC-CBF, and (**d**) IC-TTP.

**Fig. (4):**
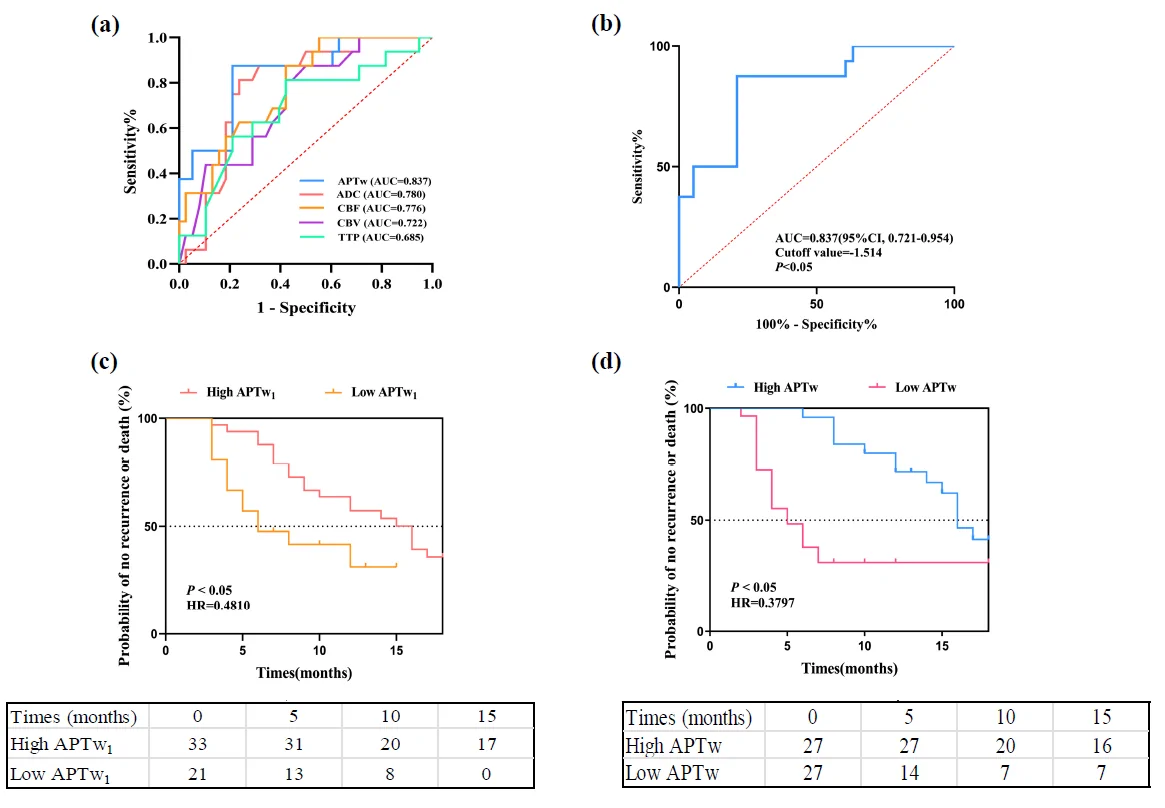
Prognostic assessment of 18-month outcomes using APTw Imaging. (**a**, **b**) ROC curve for predicting 18-month outcomes with different predictors. (**c**) Kaplan-Meier curve based on the ROC cutoff value from Figure **3b**. (**d**) Kaplan-Meier curve based on the median APTw value.

**Fig. (5a-e):**
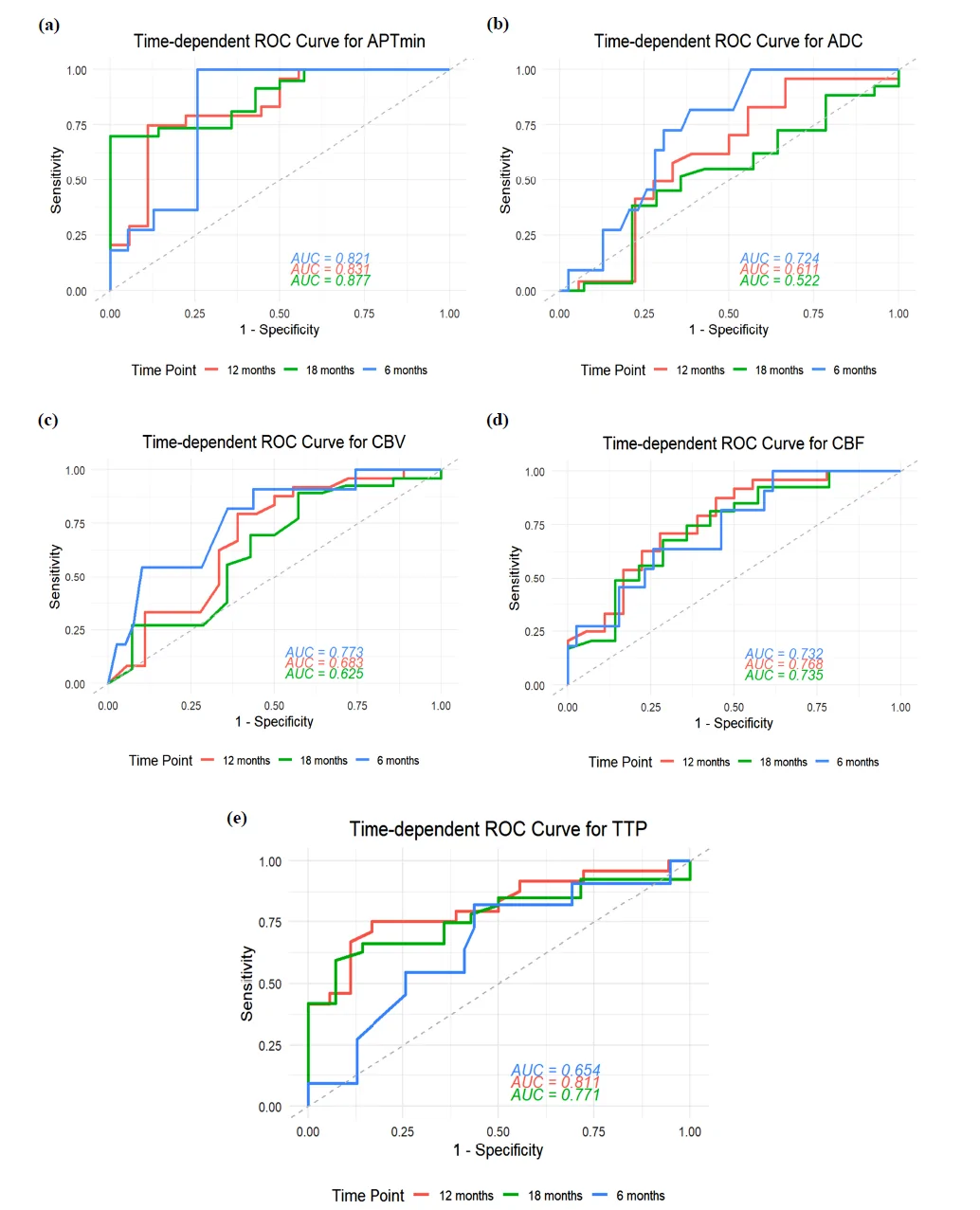
Time-dependent ROC curves for different predictors.

**Fig. (6a-c):**
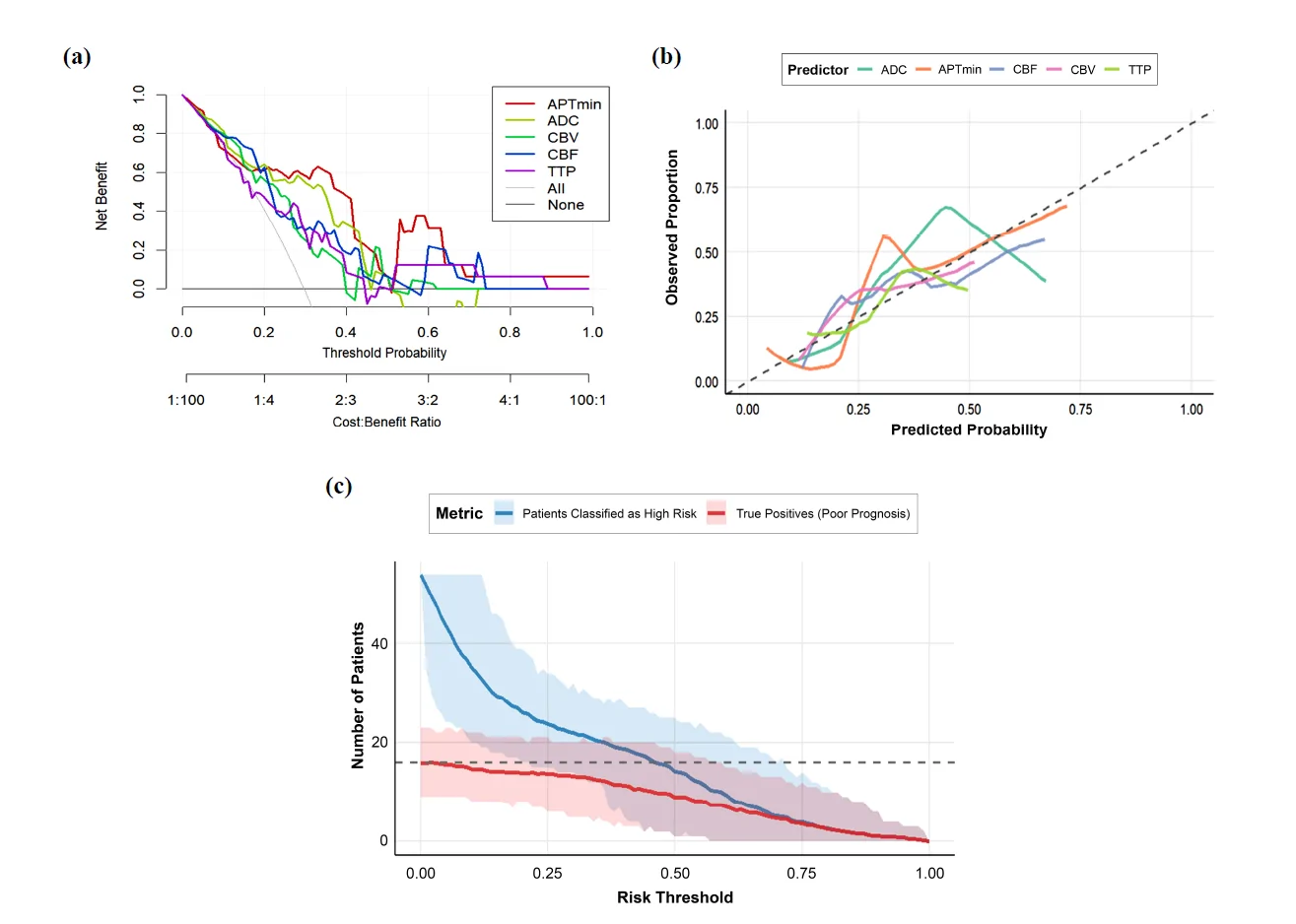
Decision Curve Analysis (DCA), calibration curves, and Clinical Impact Curve (CIC) of different predictors.

**Fig. (7):**
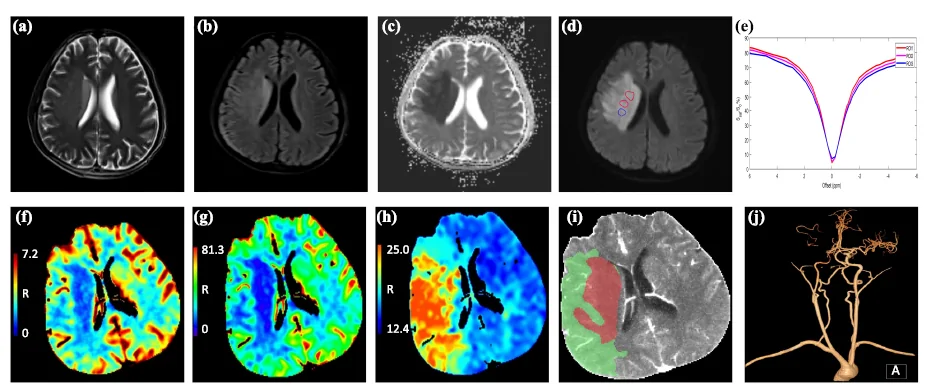
A 40-year-old male with acute ischemic stroke, admitted after a fall, presenting with an NIHSS score of 11, and no recurrence at 18-month follow-up.(**a**) T2WI. (**b**) FLAIR. (**c**) ADC map showing low signal within the infarcted area (mean ADC = 0.465 × 10^−3^ mm^2^/sec). (**d**) DWI demonstrated acute infarction in the right basal ganglia and paraventricular region. (**e**) Z-spectrum. (**f**) CBV. (**g**) CBF. (**h**) TTP. (**i**) Mismatch between CBF and Tmax. (**j**) VR revealed occlusion of the distal right internal carotid artery (C2), right anterior cerebral artery (A1), and middle cerebral artery (M1) with sparse distal branching.

**Fig. (8):**
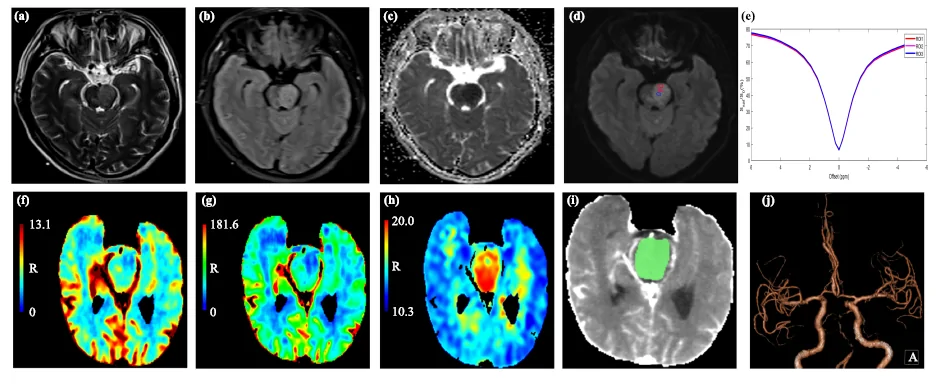
A 51-year-old male with acute ischemic stroke, admitted for unconsciousness, presenting with an NIHSS score of 17, and deceased 2 months after discharge.(**a**) T2WI. (**b**) FLAIR. (**c**) ADC map showing low signal within the infarcted area (mean ADC = 0.445 × 10^−3^ mm^2^/sec). (**d**) DWI demonstrated acute infarction in the brainstem. (**e**) Z-spectrum. (**f**) CBV. (**g**) CBF. (**h**) TTP. (**i**) Mismatch between CBF and Tmax. (**j**) VR demonstrated focal occlusion of the distal basilar artery, accompanied by multiple severe luminal stenoses.

**Table 1:** Baseline information for patients with different mrs scores.

-	mRS(0-2)	mRS(3-6)	Statistic	*P*
**Sex (F: M)**	8:29	5:12	0.78	0.378
**Age**	62(55.75,69.25)	69(59,76)	-1.92	0.055
**LDL**	3.00±0.86	2.67±0.95	1.34	0.186
**Lesion length**	21.5(11.98,26.55)	34.60(25.00,48.00)	-3.33	<0.01**
**ADC**	0.48(0.45,0.53)	0.43(0.41,0.45)	-4.06	<0.001***
**NIHSS**	6.24±3.39	13.70±3.38	-8.35	<0.001***
**APT_min_**	-1.42(-1.56,-1.23)	-1.61(-1.63,-1.52)	-4.20	<0.001***
**Smoking**	30 (81.1%)	13 (76.5%)	0.001	0.978
**Hypertension**	35 (94.6%)	15 (88.2%)	0.07	0.788
**Diabetes**	27 (73.0%)	14 (82.4%)	0.17	0.685
**IC-CBV**	3.20±1.16	2.26±0.78	3.77	<0.001***
**IC-CBF**	36.12±10.15	20.98±9.32	5.94	<0.001***
**IC-TTP**	18.10(14.33,20.43)	21.60(19.20,22.80)	-2.82	<0.01**
**IC-MTT**	7.30(5.00,9.90)	8.60(7.30,10.50)	-1.57	0.117
**IC-T_max_**	9.20(2.70,12.40)	11.80(9.00,12.80)	-1.54	0.123

**Abbreviations:** mRS: Modified Rankin Scale; F: female; M: male; LDL: low density lipoprotein, mmol/L; Lesion size: maximum length of the lesion, mm; ADC: apparent diffusion coefficient, *10^-3^mm^2^/s; NIHSS: National Institute of Health stroke scale; APTmin: amide proton transfer weight minimum; IC: infarct core; CBF: cerebral blood flow, ml/100g/min; CBV: cerebral blood volume, ml/100g; MTT: mean transit time, /s; TTP: time to peak, /s; Tmax: time to max enhancement, /s; **indicates statistically significant (*p* <0.01). *** Indicates statistically significant (*p* <0.001).

## Data Availability

The data and materials used in this study are available upon reasonable request by the corresponding authors [Y.L.], [X.Z] and [X.Z].

## LIST OF ABBREVIATIONS

AUC: Area under the curve
CT: Computed tomography
APTw: Amide Proton Transfer – weighted
ADC: Apparent diffusion coefficient
CTP: Computed tomography perfusion
KM: Kaplan and Meier
NIHSS: National Institutes of Health Stroke Scale
mRS: Modified Rankin Scale
ICC: Intraclass correlation coefficient
CBV: Cerebral blood volume
CBF: Cerebral blood flow
TTP: Time-to-peak
ROC: Receiver operating characteristic curve
DCA: Decision curve analysis
CIC: Clinical impact curves

## STANDARDS OF REPORTING

STROBE guidelines were followed.
