# Supplementary material for: Clinical Value of Amide Proton Transfer-weighted Imaging in Ischemic Stroke: A Comparative Study with Apparent Diffusion Coefficient and CT Perfusion
Source: Curr Med Imaging. 2026 Aug 27;22:E15734056493222. doi: 10.2174/0115734056493222260804100143 (PMC13741133; doi:10.2174/0115734056493222260804100143)
Supplement: Supplementary file 1 [file CMIM-22-E15734056493222_SD1.pdf]

1875-6603/26

Send Orders for Reprints to reprints@benthamscience.net

BENTHAM SCIENCE

CrossMark

Current Medical Imaging

Content list available at: https://benthamscience.com/journals/cmir

Supplementary Material

Clinical Value of Amide Proton Transfer-weighted Imaging in Ischemic Stroke: A Comparative Study with Apparent Diffusion Coefficient and CT Perfusion

Le Zhou[1,#], Renjun Huang[1,#], Hui Wang[2,#], Mengying Zhu[1], Xiaofei Zhou[1,*], Yi Zhang[3], Xinbing Zheng[4,*] and Yonggang Li[1,5,*]

[1]*Department of Radiology, The First Affiliated Hospital of Soochow University, Suzhou City, Jiangsu Province, 215006, China*
[2]*Department of Neurology, The First Affiliated Hospital of Soochow University, Suzhou, Jiangsu, 215000, China*
[3]*Key Laboratory for Biomedical Engineering of Ministry of Education, Department of Biomedical Engineering, College of Biomedical Engineering and Instrument Science, Zhejiang University, Hangzhou, Zhejiang, China*
[4]*Department of Radiology, Zhangjiagang Sixth People's Hospital, Suzhou City, Jiangsu Province, 215600, China*
[5]*Institute of Medical Imaging, Soochow University, Suzhou City, Jiangsu Province, 215000, China*

Table S1. ROC performance of imaging biomarkers for predicting poor functional outcome.

Biomarker	AUROC	95% CI
APTmin	0.877	0.756–0.967
ADC	0.522	0.374–0.679
CBV	0.625	0.461–0.764
CBF	0.735	0.550–0.842
TTP	0.771	0.631–0.910

© 2026 The Author(s). Published by Bentham Science Publisher.

This is an open access article distributed under the terms of the Creative Commons Attribution 4.0 International Public License (CC-BY 4.0), a copy of which is available at: https://creativecommons.org/licenses/by/4.0/legalcode. This license permits unrestricted use, distribution, and reproduction in any medium, provided the original author and source are credited.

HOW TO CITE:
Zhou L, Huang R, Wang H, Zhu M, Zhou X, Zhang Y, Zheng X, Li Y. Clinical Value of Amide Proton Transfer-weighted Imaging in Ischemic Stroke: A Comparative Study with Apparent Diffusion Coefficient and CT Perfusion. Curr Med Imaging, 2026; 22: e15734056493222. http://dx.doi.org/10.2174/0115734056493222260804100143
